# Correction: Synergistic bimetallic Fe–Cu@hydrochar composite for efficient pharmaceutical removal *via* coupled adsorption and Fenton-like catalysis

**DOI:** 10.1039/d6ra90090a

**Published:** 2026-07-30

**Authors:** Maria Islam, Zia Ul Haq Khan, Mahmood M. S. Abdullah, Amjad Khan, Fida Ullah, Muhammad Idrees

**Affiliations:** a Department of Chemistry, COMSATS University Islamabad, Islamabad Campus Park Road 45550 Pakistan ziaulhaqkhan11@gmail.com; b Surfactants Research Chair, Department of Chemistry, College of Science, King Saud University P. O. Box 2455 Riyadh 11451 Saudi Arabia; c Institute of Biological Sciences, Department of Zoology, University of Lakki Marwat Khyber Pakhtunkhwa Pakistan; d State Key Laboratory of Chemical Resource Engineering, College of Chemistry, Beijing University of Chemical Technology Beijing 100029 China; e School of Chemistry and Chemical Engineering, Guizhou University Guiyang 550025 China

## Abstract

Correction for ‘Synergistic bimetallic Fe–Cu@hydrochar composite for efficient pharmaceutical removal *via* coupled adsorption and Fenton-like catalysis’ by Maria Islam *et al.*, *RSC Adv.*, 2026, https://doi.org/10.1039/d6ra04294e.

The authors regret that the name of one of the authors (Maria Islam) was shown incorrectly in the original article. The corrected author list is as shown above.

The Royal Society of Chemistry apologises for these errors and any consequent inconvenience to authors and readers.

